# Compound microbial intelligent membrane fermentation reshapes the bacterial community and reduces antibiotic, metal, and biocide resistance genes and virulence factors in duck litter

**DOI:** 10.3389/fmicb.2026.1997145

**Published:** 2026-10-26

**Authors:** Tiecheng Sun, Guiheng Mei, Meiqin Li, Junru Mo, Chengwei Zhang, Yanmei Ma, Jiamei Zhao, Song Peng, Fengqiang Lin, Ji-Long Chen, Zhaolong Li

**Affiliations:** 1 Key Laboratory of Animal Pathogen Infection and Immunology of Fujian Province, College of Animal Sciences, Fujian Agriculture and Forestry University, Fuzhou, China; 2 Gut Microbiome Research Center, Institute of Animal Husbandry and Veterinary Medicine, Fujian Academy of Agricultural Sciences, Fuzhou, Fujian, China

**Keywords:** antibiotic resistance genes, biocide resistance genes, duck litter, metagenomics, metal resistance genes, virulence factors

## Abstract

**Background:**

Contaminated duck litter is a leading cause of flock disease and a bottleneck for the sustainable development of large-scale dry-rearing systems.

**Methods:**

We applied shotgun metagenomics to litter from four positions within each of three independent compound microbial intelligent membrane fermentation beds, with unfermented litter from the same three batches as the control. We investigated the bacterial community alongside the abundance of antibiotic, metal and biocide resistance genes (ARGs, MRGs and BRGs) and of virulence factor genes (VFGs).

**Results:**

Fermented litter was consistently enriched in thermophilic and thermotolerant taxa and in beneficial genera such as *Paenibacillus* and *Bacillus*, while opportunistic pathogens including *Streptococcus*, *Mycobacterium* and *Escherichia* were clearly reduced. The total burden of the ARG and VFG classes fell in every fermented position, and representative determinants were consistently depleted: *floR*, *qacEdelta1* and *sul2* among the ARGs, *ruvB* among the MRGs, *mexF*, *ideR* and *sodA* among the BRGs, and *htpB*, *clpV* and *katA* among the VFGs. A small number of genes, *tet(L)*, *fexA* and *bacA* among them, rose in the most active zones, indicating structural remodeling of the resistance reservoir rather than uniform removal. Fermentation was not spatially uniform: the upper and edge layers were more active, and the center-lower layer remained closest to the unfermented control.

**Conclusion:**

Intelligent membrane fermentation reduced the overall abundance of resistance and virulence determinants in the litter microbiome, and spatial uniformity is important for the process. Because these data are DNA-based, they describe the abundance of resistance and virulence determinants and not resistance or virulence phenotypes.

## Introduction

China is the world’s largest producer of waterfowl, marketing more than four billion meat ducks a year and holding several hundred million laying ducks. That scale secures a major source of animal protein. It also concentrates environmental and disease-control pressures. Conventional waterfowl production depends heavily on open water: birds defecate directly into ponds and rivers, and the resulting nutrient loading drives eutrophication. As environmental regulation has tightened and access to water bodies has narrowed, producers have moved toward dry rearing, keeping ducks indoors or on exercise yards bedded with litter that absorbs manure and keeps the floor dry. The deep-litter fermentation bed, usually built from rice husk and sawdust and inoculated with functional microorganisms that decompose manure in situ, now dominates dry rearing and is widely used in Guangdong, Fujian and Guangxi (He et al., 2015; Chen et al., 2017; Xu, 2023).

Continuous use, however, turns the bedding into a complex contaminated matrix. Feces, shed feathers, spilled feed and water accumulate over successive batches, and in warm, humid conditions the litter becomes rich in organic matter and microorganisms. Damp, moldy, biologically contaminated litter is a recognized trigger of aspergillosis, *Riemerella anatipestifer* infection and *salmonellosis* in ducks, and the resulting disease burden raises both mortality and medication costs (Chen et al., 2017).

Ecologically, contamination of the litter reflects a shift in community structure and the accumulation of harmful functional genes. In a healthy bed, beneficial microorganisms hold competitors in check through resource competition and metabolic inhibition. Persistent manure loading, together with increasingly anaerobic and microaerophilic conditions, drives a successional replacement of that community. In meat duck fermentation beds, the bacterial communities of fresh litter and of litter used for four and eight batches were 68.81% and 70.82% similar to one another, whereas the four- and eight-batch communities were 81.93% similar (Bi et al., 2013; Zhang et al., 2015). The community, in other words, converges on a stable configuration as the bed ages. That stable state is not a healthy equilibrium. It is a community dominated by particular spoilage organisms and opportunistic pathogens under continuous manure input and selection.

Selection also comes from the farm itself. Antibiotics, disinfectants and the copper and zinc added to feed are largely unabsorbed in the gut and are excreted into the litter, where they exert sustained selective pressure. Antibiotic and heavy metal concentrations build up over successive batches, and bacterial tolerance rises in parallel (Lin et al., 2015). Metagenomic surveys of duck farm feces and litter have found very high abundances of copper and zinc resistance genes, as expected from feed supplementation (Xiong et al., 2023). One survey of duck farm environments identified 823 ARG subtypes across 18 classes, floR the most abundant, followed by sul1, tetM, sul2 and tetL (Liu et al., 2024). A longitudinal comparison of broiler and Cherry Valley duck systems showed that the duck resistome is environment-centered: ARGs unique to environmental samples outnumbered those in the fecal litter fraction by about 2.6 times, and clinically relevant opportunists such as *Pseudomonas aeruginosa, Escherichia coli, Acinetobacter baumannii* and *Staphylococcus aureus* were repeatedly detected (Fauszt et al., 2026). Biocide resistance genes and virulence factor genes (VFGs) are widespread in litter as well. Together these determinants define the litter resistome and virulome, with implications for animal health, food safety and public health.

Current responses to litter contamination are physical replacement, chemical disinfection and biological fermentation. Frequent replacement is expensive and generates secondary waste; disinfection leaves residues and can select for new resistances. Biological treatment is generally held to be more sustainable, because aerobic fermentation raises the temperature and mobilizes microbial competition against pathogens (Liu et al., 2021; Agga and Loughrin, 2026). Metagenomic sequencing has confirmed that supplementing anaerobic digestion of poultry litter with micro-aeration substantially alters the resistome and virulence gene profile (Agga and Loughrin, 2026). Conventional fermentation beds, though, ferment unevenly: performance differs markedly between zones of the same bed, and their effect on ARGs and metal resistance genes (MRGs) has rarely been assessed systematically.

Intelligent membrane fermentation offers a different route. A functional polymer membrane is laid over the material, and an automatic control system monitors and adjusts temperature and oxygen in real time to sustain thermophilic aerobic fermentation. Against open or simply covered fermentation, the approach gives more consistent temperature control, a more stable process and lower odor emissions (Ma et al., 2017; Li et al., 2021). Metaviromic work has shown that combined microbial and intelligent membrane fermentation reshapes the virome and reduces resistance genes in duck litter (Sun T. et al., 2026), and the decline of ARGs during hyperthermophilic composting is well documented (Wang et al., 2024). Even so, microbiome-level studies of intelligent membrane fermentation applied to duck litter remain scarce. Its combined effect on the bacterial community, and on ARGs, MRGs, biocide resistance genes (BRGs) and VFGs, is unresolved.

Shotgun metagenomics suits this question well. Unlike culture-based methods or 16S rRNA amplicon sequencing, it recovers DNA from all microorganisms present without primer bias, and it characterizes species composition together with the diversity and abundance of ARGs, MRGs, BRGs and VFGs from a single dataset. That is why it has become standard for resistome and virulome surveys of livestock manure and litter (Xiong et al., 2023; Liu et al., 2024; Fauszt et al., 2026; Sun T. et al., 2026). Here we applied it to duck litter fermented with a compound microbial inoculant under an intelligent membrane, sampling four positions within each of three independent fermentation beds and using unfermented litter from the same three batches as the control, so that the comparison isolates fermentation rather than any difference in source material, batch or season. We asked how fermentation alters the bacterial community, whether it enriches beneficial taxa such as Paenibacillus and Bacillus while suppressing potential pathogens such as Streptococcus, Mycobacterium and Escherichia, and how far it reduces the abundance of ARGs such as floR, qacEdelta1 and sul2, MRGs such as ruvB, and VFGs such as htpB, clpV and katA.

## Materials and methods

### Fermentation setup and process monitoring

Each bed measured 20 m in length, 7 m in width and 2 m in height and was charged with a bedding mixture of rice husk and sawdust at a ratio of 6:4 (v/v) to a loading depth of 70 cm. The bedding was inoculated with a compound microbial fermentation agent developed by the Institute of Animal Husbandry and Veterinary Medicine, Fujian Academy of Agricultural Sciences (Fuzhou, China) and containing *Bacillus subtilis, Bacillus licheniformis, Lactobacillus plantarum* and *Saccharomyces cerevisiae* at a viable count of ≥ 1 × 10^9^ CFU/g, applied at 0.1% (w/w) of the bedding, equivalent to about 1 kg per tonne, and mixed thoroughly with the bedding on the day the material was loaded. The inoculated material was then covered with a functional semi-permeable polymer membrane (expanded polytetrafluoroethylene, 0.3 mm thick; Qingdao Huanong Innovation Technology Co., Ltd., Qingdao, China) and fermentation was continued for 21 d.

The beds were not turned at any time during fermentation; the material was aerated only. Air was supplied through perforated pipes laid along the bottom of each bed, in an intermittent cycle of 30 min on and 60 min off, two to four cycles per day, at an air flow of about 0.2 L min^−1^ m^−3^ of bedding. Temperature and oxygen were monitored throughout fermentation with PT100 temperature probes and electrochemical oxygen sensors placed at each of the four sampling positions and recorded every 30 min. Temperature differed markedly with depth and position: the highest temperature recorded was 85 °C at 60 cm below the surface, and the bed remained above 55 °C for 7 d, whereas the edge reached only 56 °C. Oxygen averaged 16–18% in the upper and edge layers and 10–12% in the deeper central litter. The initial moisture content was 55% and the initial C/N ratio was 25:1. These records document that thermophilic conditions were established throughout the bed, most strongly in the deeper central litter, while the oxygen supply differed among the four sampling positions.

### Sample collection

Litter was sampled from three independent fermentation beds operated in parallel on a commercial duck farm in Fujian Province, China. Within each bed, four positions were sampled: center-lower (DLZX), center-upper (DLZS), edge-lower (DLBX), and edge-upper (DLBS). Unfermented litter served as the control (CK). Each group comprised three replicates, one from each of the three independent beds. The center positions were taken at the middle of the bed length and the edge positions within 1 m of the side wall; upper denotes the layer 0–20 cm below the surface and lower the layer 40–70 cm below the surface. The control was the same bedding material – the rice husk and sawdust base carrying the same compound microbial inoculant – taken from the same three batches immediately before the material was loaded into the beds and covered with the membrane. The samples were transported on ice to the laboratory and stored at -80 °C until DNA extraction. Sampling was carried out on day 21, at the end of the fermentation period. Each bed was sampled, library-prepared and sequenced independently, and the values reported for a position are the means of the three beds. The four sampling positions are not treated anywhere in this study as independent replications of the fermentation treatment; comparisons among them are within-bed spatial contrasts and are interpreted as such. Each of the five groups therefore contains three independent biological replicates, giving 15 samples in total, and the bed is the experimental unit for every comparison reported below.

### DNA extraction and sequencing

Microbial DNA was extracted from the litter samples with the E.Z.N.A.^®^ Stool DNA Kit (Omega Bio-tek, Norcross, GA, USA) following the manufacturer’s instructions. Library construction and sequencing were performed by Shanghai Lingen Biotechnology Co., Ltd. (Shanghai, China). Briefly, 1 μg of genomic DNA per sample was fragmented to approximately 450 bp with a Covaris S220 focused-ultrasonicator (Woburn, MA, USA) and used to build sequencing libraries, which were sequenced on an Illumina NovaSeq 6000 platform in paired-end 150 bp (PE150) mode. Raw reads were trimmed of adapters and low-quality bases with Trimmomatic (Bolger et al., 2014). Quality-filtered reads were then mapped back to the host genome with BWA-MEM (-M -k 32 -t 16) to remove host contamination and residual low-quality data; the resulting clean data were used for all downstream analyses.

### *De novo* assembly, gene prediction, and annotation

Clean reads from each sample were assembled with MEGAHIT (–min-contig-len 500) (Li et al., 2015) to generate contigs. Open reading frames were predicted and the assembly was dereplicated with CD-HIT (-n 9 -c 0.95 -G 0 -M 0 -d 0 -aS 0.9 -r 1) (Fu et al., 2012) to obtain a non-redundant (unique) gene set, with the longest sequence in each cluster taken as the cluster representative. Gene abundance in each sample was estimated from read counts obtained with Salmon (Patro et al., 2017) using (Equations 1– 4):


A⁢b⁢(S)=A⁢b⁢(U)+A⁢b⁢(M) (1)

$$\text{Ab}\,\left(\text{U}\right)=\overset{\text{M}}{\underset{\text{i}=1}{\sum }}1/l$$ (2)

$$A\,b\,\left(M\right)=\overset{M}{\underset{i=1}{\sum }}\left(C\,o*1\right)/l$$ (3)

$$\text{Co}=\frac{\mathrm{Ab}\,\left(U\right)}{\sum_{\text{i}=1}^{\text{N}}\mathrm{Ab}\,\left({\text{U}}_{\text{i}}\right)}$$ (4)

where *Ab(S)* is gene abundance, *Ab(U)* is the abundance of uniquely mapped reads, *Ab(M)* is the abundance of multi-mapped reads, and *l* is gene length (Li et al., 2014).

The unique gene set was aligned against the KEGG database with BLASTX to assign protein functions, and pathway maps were drawn from the KO annotations of the 15 samples. Predicted genes were then translated and aligned against the Carbohydrate-Active enZYmes database (CAZy) (Lombard et al., 2014) and the eggNOG database (Huerta-Cepas et al., 2016) for functional annotation, with alignments performed by DIAMOND (Buchfink et al., 2015).

### Annotation of ARGs, MRGs, BRGs and VFGs

This study focuses on antibiotic, metal and biocide resistance genes and virulence factor genes, annotated as follows. Predicted protein sequences from the non-redundant gene catalogue were aligned with DIAMOND against three curated databases. ARGs were identified against SARG v2.2 (Yin et al., 2018), MRGs and BRGs against the experimentally confirmed subset of BacMet 2.0 (Pal et al., 2014), and VFGs against the 2022 release of the VFDB core dataset (Liu et al., 2022). For each database, the best hit was retained, and a gene was assigned only when identity was ≥80%, query coverage was ≥70% and the E value was ≤ 1 × 10^−5^. Gene abundance was calculated as RPKM, normalized to gene length and total clean reads, then summed by database hierarchy into resistance mechanisms, functional categories and antibiotic/metal/biocide classes. Total burden per class was the sum of the abundances of the subtypes assigned to it, reported as the total ARG, MRG, BRG or VFG burden.

### Metagenome-assembled genomes and abundance estimation

Contigs from each sample were binned with metaBAT2 (Kang et al., 2015). Bin quality was assessed with CheckM v1.0.3 (Parks et al., 2015), and bins with completeness >50% and contamination <10% were retained. To improve MAG quality, the retained bins were reassembled with metaSPAdes (Nurk et al., 2017), the corresponding reads were extracted from the clean data with BWA-MEM, and MAG abundance was calculated with the quant_bins module of MetaWRAP using default parameters. Because CheckM includes single-copy gene sets at different phylogenetic levels, the most similar level was assigned as the phylogenetic annotation of each bin by comparing bin genes with the reference genes at each level. The proteome of each bin was derived by translating all bin genes and was used to reconstruct a phylogenetic tree with PhyloPhlAn (Segata et al., 2013). MAGs were reconstructed to place the dominant populations in a genomic context and to support the taxonomic interpretation of the community data presented in this paper.

### Alpha and beta diversity analysis

Beta diversity was analysed with the community ecology package vegan in R using a Bray–Curtis distance matrix (Oksanen et al., 2020) and 999 permutations. Principal component analysis (PCA), principal coordinates analysis (PCoA), and non-metric multidimensional scaling (NMDS) were also performed with vegan. Differences in community structure among the five groups were tested by permutational multivariate analysis of variance (PERMANOVA) using adonis2 with 999 permutations; the explained variance (*R*^2^ = 0.919), the pseudo*-F* statistic (pseudo*-F* = 28.41) and the permutation *P*-value (*P* = 0.001) are reported (Anderson, 2001).

### Statistical analysis

All analyses were performed in R (v4.3.1) and GraphPad Prism (v9.0). Normality was tested with the Shapiro–Wilk test and homogeneity of variance with Levene’s test. Groups were compared by one-way analysis of variance (ANOVA) followed by Tukey’s HSD test for all pairwise comparisons, or, where an assumption was not met, by the Kruskal–Wallis test followed by Dunn’s test. Because several hundred taxa and ARG, MRG, BRG and VFG features and their resistance classes were compared in parallel, these comparisons are exploratory: the *P*-values are reported without correction for multiple testing, and a feature was considered significantly different at *P* < 0.05. Significance is indicated as *P < 0.05, **P < 0.01 and ***P < 0.001, or with the letters a (*P* < 0.05), aa (*P* < 0.01) and aaa (*P* < 0.001) for comparison with the unfermented control. All statistical tests were performed on the 15 samples, which provide three independent biological replicates per group with the fermentation bed as the experimental unit. All shotgun metagenomic analyses were performed on the non-redundant gene catalogue described above; amplicon-based taxonomic normalization and metabolite profiling were not part of this study.

## Results

### Alpha and beta diversity of the litter bacterial community

Alpha diversity describes species richness and diversity within a sample. Richness varied among the groups. The Chao1 index separated DLBS from both CK and DLZX (*P* < 0.05), and the Shannon index followed the same pattern, again distinguishing DLBS from CK and from DLZX (*P* < 0.05). Simpson showed nothing: it did not differ among groups (Figures 1A–C).

**FIGURE 1:**
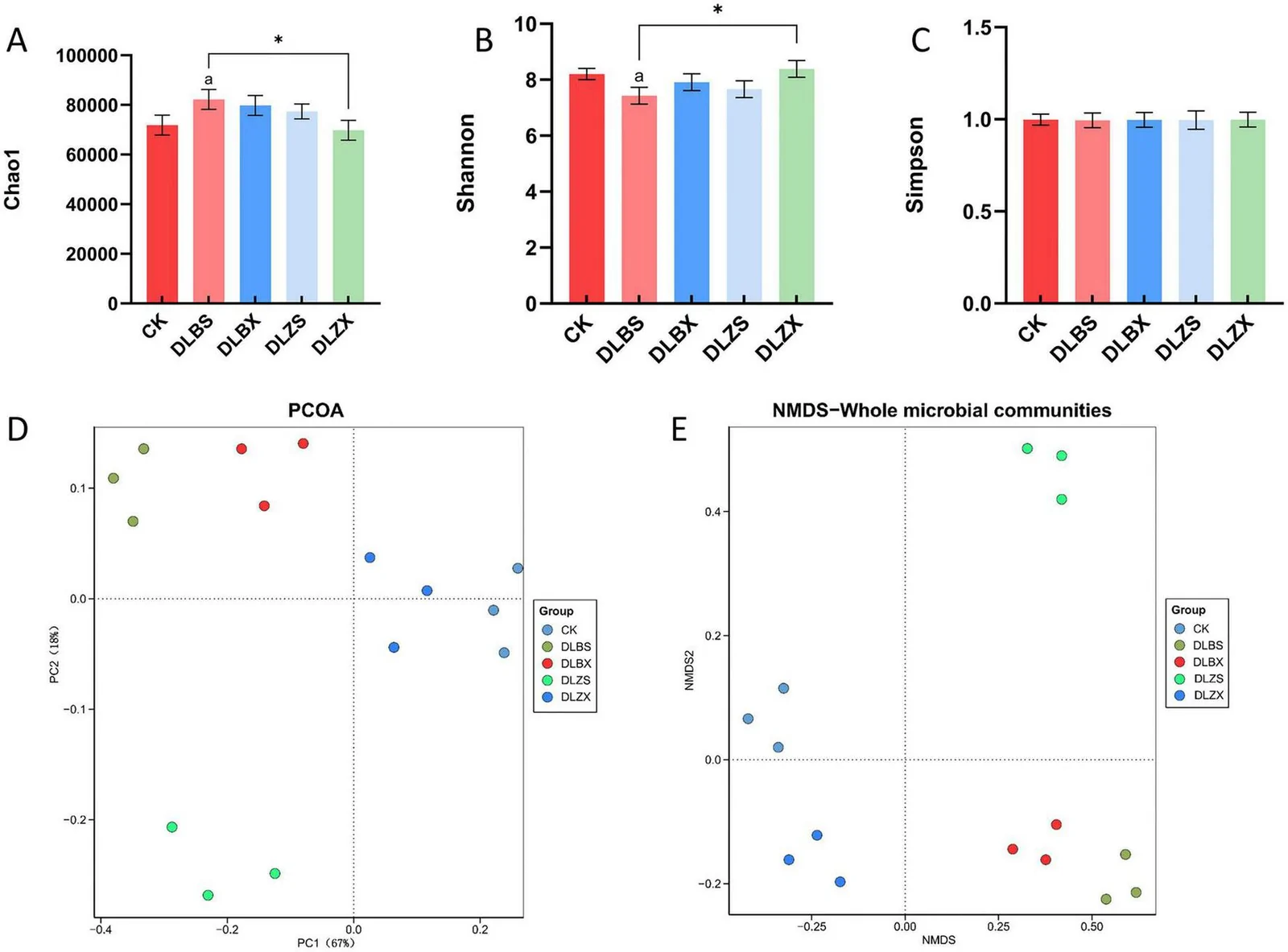
Alpha and beta diversity of the bacterial community in duck litter. **(A–C)** Alpha diversity indices: **(A)** Chao1, **(B)** Shannon, **(C)** Simpson. **(D, E)** Beta diversity: **(D)** PCoA, in which PC1 and PC2 explained 67% and 18% of the variation; **(E)** NMDS. In this figure, a denotes a significant difference from the control (*P* < 0.05); unlabeled comparisons were not significant.

Beta diversity separated the communities clearly. PCoA and NMDS both resolved significant differences in structure among groups, though the spread within groups was visible. PC1 and PC2 together accounted for 85% of the variation (67% and 18%). PERMANOVA on Bray–Curtis distances confirmed that group identity explained a significant share of the variation in community structure (R^2^ = 0.919, pseudo-F = 28.41, *P* = 0.001; 999 permutations) (Figures 1D, E).

### Bacterial community composition phylum level

The ten most abundant phyla were plotted and the core differential taxa analysed. Two phyla, *Pseudomonadota* and *Actinomycetota*, dominated all five groups. Fermentation tilted the balance between them. Against CK, *Pseudomonadota* and *Bacillota* were more abundant across all four fermented treatments, while *Actinomycetota* and *Bacteroidota* were scarcer. Where those shifts were largest differed by position: DLZS held the most *Pseudomonadota* and *Bacillota* and the least *Actinomycetota*, whereas *Bacteroidota* peaked in DLZX (Figure 2A).

**FIGURE 2:**
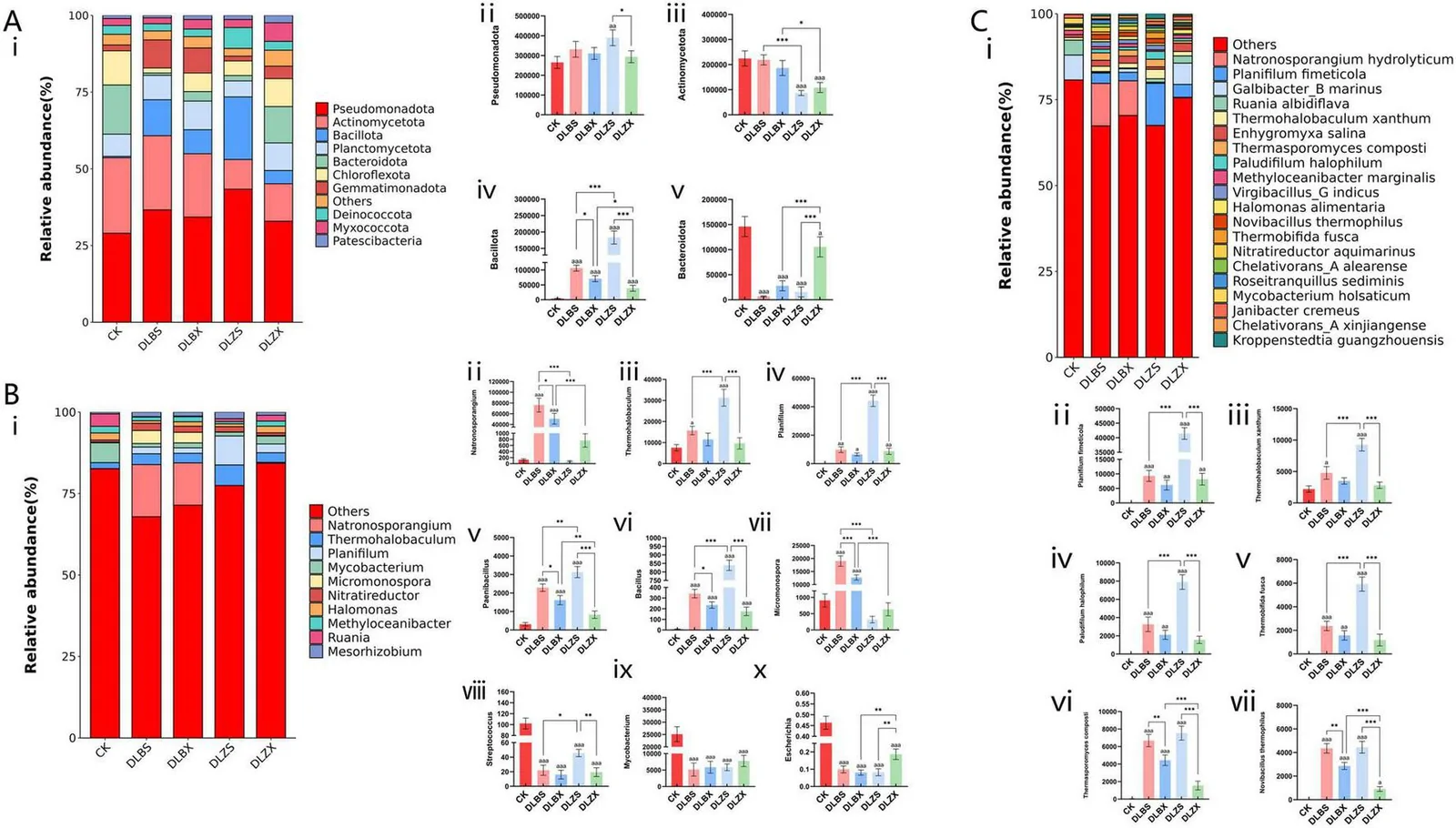
Bacterial composition, relative abundance, and between-group differences in duck litter. **(A)** Relative abundance and between-group differences at the phylum level. **(B)** Relative abundance and between-group differences at the genus level. **(C)** Relative abundance and between-group differences at the species level. *P < 0.05, **P < 0.01, ***P < 0.001.

The tests sharpened the picture. *Bacillota* was enriched in all four fermented groups (*P* < 0.001) and *Bacteroidota* depleted in all four (*P* < 0.05). *Pseudomonadota* rose only in DLZS (*P* < 0.01). *Actinomycetota* fell in DLZS and DLZX (*P* < 0.001).

Contrasts within the fermented treatments were just as pronounced. DLBX held less *Bacillota* than DLBS (*P* < 0.05). DLZS carried less *Actinomycetota* (*P* < 0.001) and more *Bacillota* than DLBS (*P* < 0.001). Passing from DLBX to DLZX, *Actinomycetota* and *Bacillota* both declined (*P* < 0.05) while *Bacteroidota* increased (*P* < 0.001). Much the same held between the two center positions, where *Pseudomonadota* and *Bacillota* were lower in DLZX (*P* < 0.05) and *Bacteroidota* higher (*P* < 0.001).

### Genus level

Composition differed markedly among groups at the genus level. Four genera, *Thermohalobaculum*, *Planifilum*, *Paenibacillus* and *Bacillus*, were more abundant in every fermented treatment than in CK, while *Streptomyces*, *Mycobacterium* and *Escherichia* were scarcer in every one. Selection against them was unambiguous: *Streptomyces*, *Mycobacterium* and *Escherichia* were depleted in all four fermented groups (*P* < 0.001) (Figure 2B).

Which beneficial genera responded, and where, depended on position. DLBS and DLBX gained Natronosporangium, Planifilum, Paenibacillus, Bacillus and Micromonospora (*P* < 0.001), with Thermohalobaculum also enriched in DLBS (*P* < 0.05). DLZS gained Thermohalobaculum, Planifilum, Paenibacillus and Bacillus (*P* < 0.001). Only Planifilum and Bacillus responded in DLZX (*P* < 0.01).

Comparisons among the fermented treatments separated the zones further. DLBX was poorer than DLBS in *Natronosporangium*, *Paenibacillus*, *Bacillus* and *Micromonospora* (*P* < 0.05). DLZS lost *Natronosporangium* and *Micromonospora* relative to DLBS (*P* < 0.001) yet gained *Thermohalobaculum*, *Planifilum*, *Paenibacillus*, *Bacillus* and *Streptomyces* (*P* < 0.001). Against DLBX, DLZX held less *Natronosporangium*, *Paenibacillus* and *Micromonospora* and more *Escherichia* (both *P* < 0.01). The same two directions appeared against DLZS: *Thermohalobaculum*, *Planifilum*, *Paenibacillus*, *Bacillus* and *Streptomyces* were all lower in DLZX (*P* < 0.001), and *Escherichia* higher (*P* < 0.01).

### Species level

The 15 most abundant species were plotted. Six of them, *Planifilum fimeticola*, *Thermohalobaculum xanthum*, *Thermasporomyces composti*, *Paludifilum halophilum*, *Novibacillus thermophilus* and *Thermobifida fusca*, were more abundant in all four fermented treatments than in CK. Five of the six, all but *T. xanthum*, were enriched in DLBS, DLBX and DLZS (*P* < 0.01), and *T. xanthum* in DLBS and DLZS (*P* < 0.05). DLZX responded least: only *P. fimeticola* and *N. thermophilus* were enriched there (*P* < 0.05).

Position again separated the fermented treatments. DLBX had less *T. composti* and *N. thermophilus* than DLBS (*P* < 0.01), while DLZS had more *P. fimeticola*, *T. xanthum*, *P. halophilum* and *T. fusca* than DLBS (*P* < 0.001). DLZX was the poorest of all. It held less *T. composti* and *N. thermophilus* than DLBX (*P* < 0.001), and less of all six species than DLZS (*P* < 0.001) (Figure 2C).

### Antibiotic resistance genes

The heatmap of resistance mechanisms showed four of the six categories, namely antibiotic target alteration, target protection, target replacement and enzymatic inactivation, at significantly lower abundance in DLBS, DLBX and DLZX than in CK (*P* < 0.05). Mutation fell in DLBS (*P* < 0.05). In DLBX both efflux pump and mutation were reduced (*P* < 0.05), and in DLZS enzymatic inactivation was (*P* < 0.05). Set against DLBS, all six mechanisms were more abundant in DLZS (*P* < 0.05). DLZX exceeded DLBX in target alteration and enzymatic inactivation (*P* < 0.05) yet fell below DLZS in the other five (*P* < 0.05) (Figure 3A).

**FIGURE 3:**
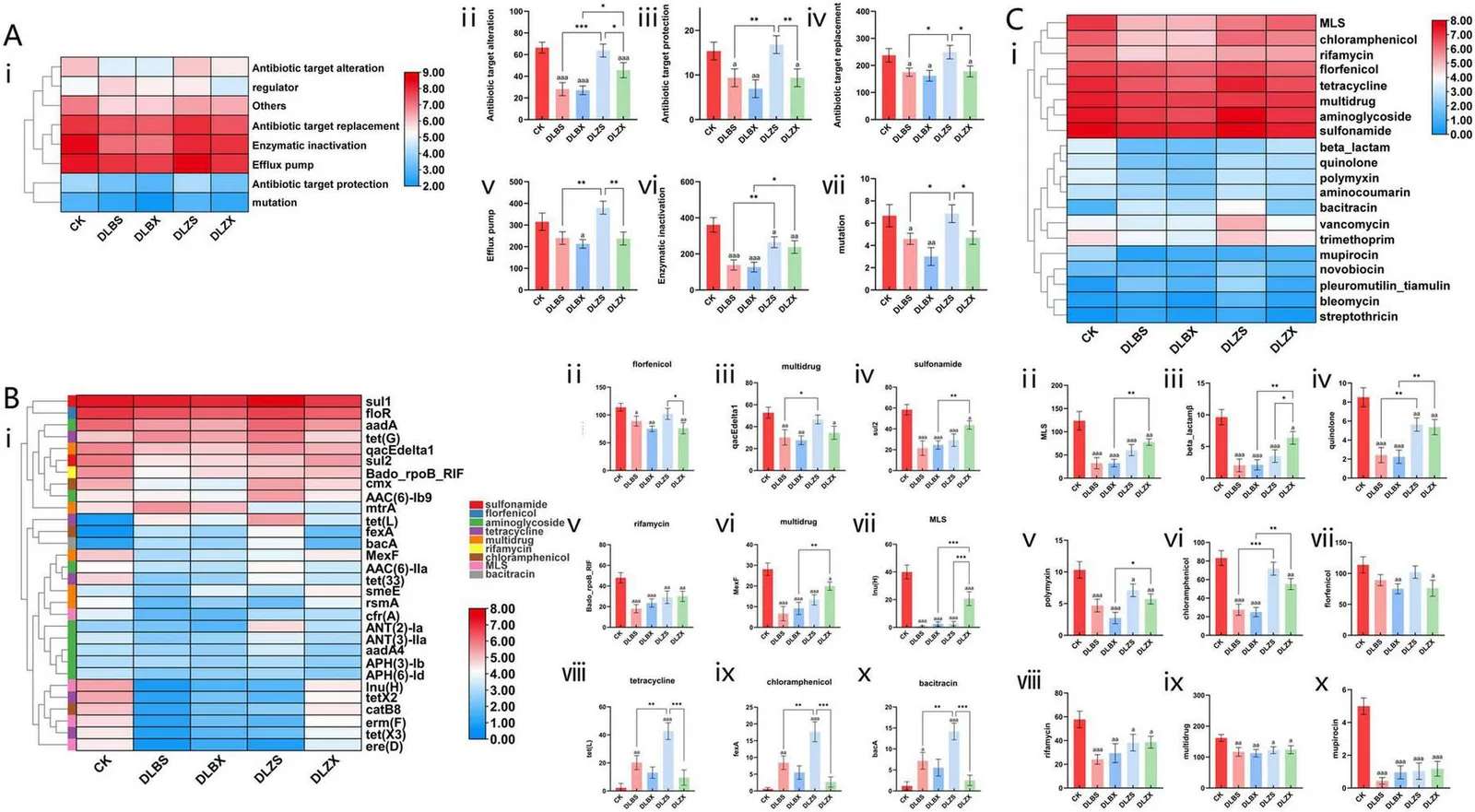
Heatmaps of antibiotic resistance genes and between-group differences. **(A)** Antibiotic resistance mechanisms. **(B)** ARG subtypes. **(C)** Antibiotic classes. *P < 0.05, **P < 0.01, ***P < 0.001.

Six subtypes declined together. floR, qacEdelta1, sul2, Bado_rpoB_RIF, MexF and lnu(H) were all lower in DLBS, DLBX and DLZX than in CK (*P* < 0.05), and sul2, Bado_rpoB_RIF, MexF and lnu(H) were lower in DLZS as well (*P* < 0.01). Against that trend, tet(L), fexA and bacA were enriched in DLBS and DLZS (*P* < 0.05) (Figure 3B).

The remaining contrasts ran in both directions. DLZS gained *qacEdelta1*, *tet(L)*, *fexA* and *bacA* relative to DLBS (*P* < 0.05). DLZX gained *sul2*, *MexF* and *lnu(H)* relative to DLBX (*P* < 0.01). Between the two center positions the subtypes parted company: *floR*, *tet(L)*, *fexA* and *bacA* were lower in DLZX (*P* < 0.05), while *lnu(H)* was higher (*P* < 0.001).

By antibiotic class, the deepest and most consistent losses were in MLS, beta-lactam, quinolone, polymyxin, rifamycin, multidrug and mupirocin, all of which were lower in every fermented group than in CK (*P* < 0.001). Chloramphenicol and florfenicol fell in DLBX and DLZX (*P* < 0.01), and chloramphenicol alone in DLBS (*P* < 0.001). DLZS was depleted in quinolone and chloramphenicol relative to DLBS (*P* < 0.01). Against DLBX, DLZX lost MLS, beta-lactam, quinolone, polymyxin and chloramphenicol (*P* < 0.05); against DLZS it lost only beta-lactam (*P* < 0.05) (Figure 3C).

### Total burden of resistance and virulence determinants

Before considering individual genes, we compared the summed relative abundance of all subtypes within each determinant class, which gives a direct measure of the total burden. Relative to unfermented litter, the total ARG burden was lower in all four fermented positions, by 38.9% (DLBS), 44.7% (DLBX), 3.9% (DLZS) and 29.7% (DLZX). The change was smaller and not unidirectional for the total MRG burden (-19.1% to +18.4%) and the total BRG burden (-10.0% to +21.8%), whereas the total VFG burden fell in all four positions (-26.5% to -39.5%) (Table 1). The reduction was therefore partial and selective: the ARG and VFG pools shrank in every position, while individual MRG and BRG classes were more abundant in some positions than in the control, and those increases are reported explicitly in the sections that follow (Figure 2B).

**TABLE 1:** Total burden of resistance and virulence determinants in unfermented litter (CK) and in the four fermented bed positions.

Determinant class	Subtypes (n)	CK	DLBS (edge-upper)	DLBX (edge-lower)	DLZS (center-upper)	DLZX (center-lower)
ARGs	241	1159.1 (–)	708.6 (-38.9%)	641.2 (-44.7%)	1114.3 (-3.9%)	815.2 (-29.7%)
MRGs	136	807.8 (–)	858.9 (+6.3%)	755.0 (-6.5%)	956.6 (+18.4%)	653.5 (-19.1%)
BRGs	96	493.3 (–)	489.4 (-0.8%)	462.2 (-6.3%)	601.0 (+21.8%)	443.9 (-10.0%)
VFGs	480	2153.5 (–)	1582.7 (-26.5%)	1423.8 (-33.9%)	1311.4 (-39.1%)	1302.6 (-39.5%)

Values are the summed relative abundances (RPKM) of all subtypes assigned to each determinant class; values in parentheses are the change relative to unfermented litter (CK). ARGs, antibiotic resistance genes; MRGs, metal resistance genes; BRGs, biocide resistance genes; VFGs, virulence factor genes. DLBS, edge-upper; DLBX, edge-lower; DLZS, center-upper; DLZX, center-lower.

### Metal resistance genes

Among the MRG subtypes the responses were mixed. *ruvB* was lower in DLBS, DLBX and DLZS than in CK (*P* < 0.01), whereas *actR*, *actP* and *hmrR* were enriched (*P* < 0.01). *acn* fell in DLBX, DLZS and DLZX (*P* < 0.01), and *merA* in DLBS (*P* < 0.05). The copper genes behaved differently again: *copP* and *copA* were enriched in DLZS (*P* < 0.001) (Figure 4A).

**FIGURE 4:**
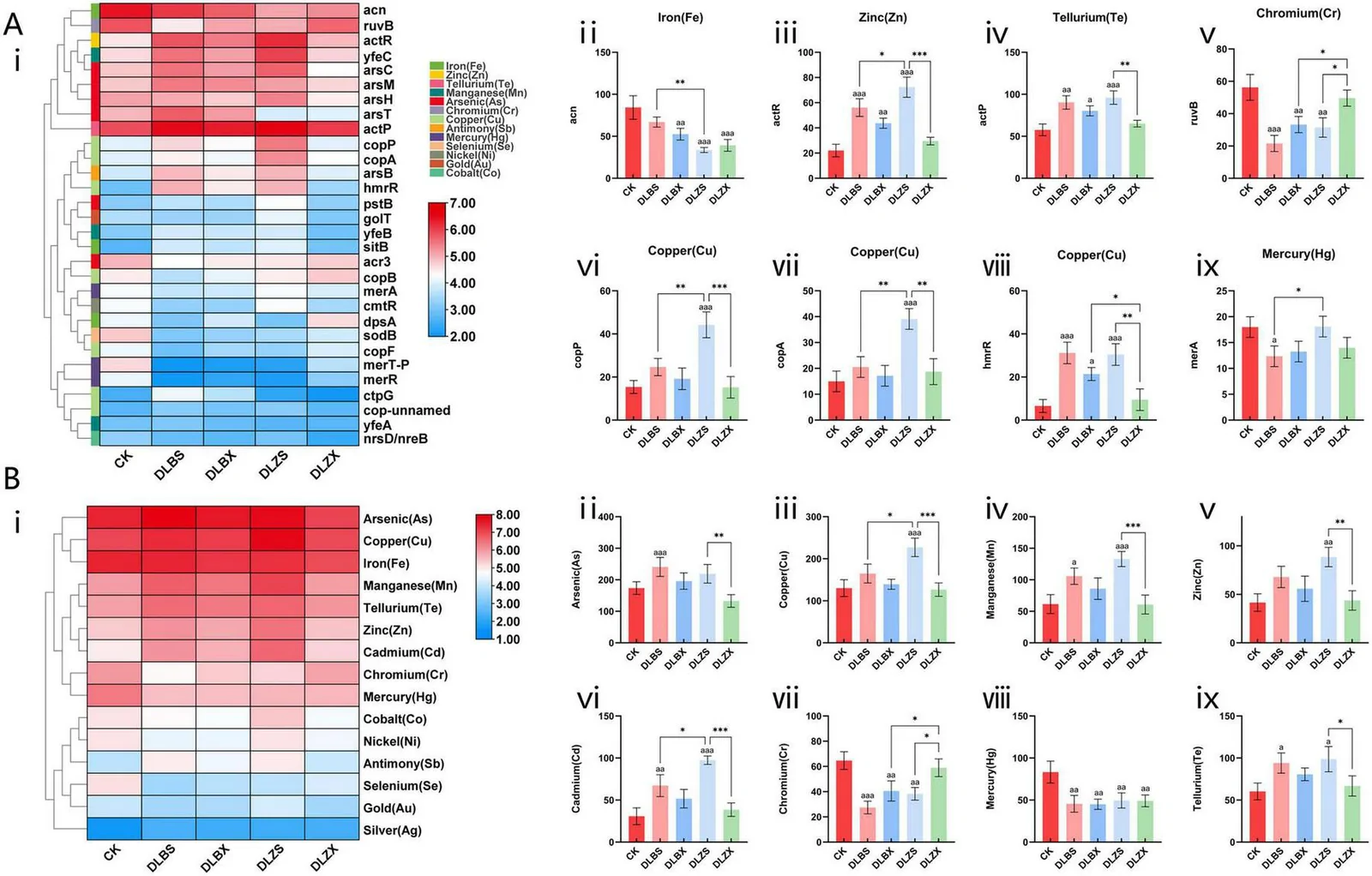
Heatmaps of metal resistance genes and between-group differences. **(A)** MRG subtypes. **(B)** Metal classes. *P < 0.05, **P < 0.01, ***P < 0.001.

Position drove the rest of the differences. DLZS lost acn relative to DLBS (P < 0.01) but gained merA, actR, copP and copA (*P* < 0.05). DLZX gained ruvB and lost hmrR relative to DLBX (both P < 0.05). Against DLZS, DLZX gained ruvB (*P* < 0.05) and lost actR, actP, copP, copA and hmrR (*P* < 0.01) (Figure 4B).

By metal class, arsenic, manganese, cadmium and tellurium were enriched in DLBS relative to CK (*P* < 0.05). DLZS accumulated five: copper, manganese, zinc, cadmium and tellurium (*P* < 0.01). Chromium and mercury moved the other way, both depleted in DLBS, DLBX and DLZS (*P* < 0.01), with mercury also depleted in DLZX (*P* < 0.05). DLZS carried more copper and cadmium than DLBS (*P* < 0.05), and DLZX more chromium than DLBX (*P* < 0.05). The widest gap lay between the two center positions: relative to DLZS, DLZX lost arsenic, copper, manganese, zinc, cadmium and tellurium (*P* < 0.01) and gained chromium (*P* < 0.05) (Figure 4B).

### Biocide resistance genes

Eight BRG subtypes, *ruvB*, *mtrD*, *sodA*, *sdeB*, *ideR*, *sodB*, *mexF* and *fabV*, were less abundant in DLBS, DLBX and DLZS than in CK (*P* < 0.001). Four of them, *sodA*, *ideR*, *sodB* and *mexF*, were also lower in DLZX (*P* < 0.01). DLZS carried more *fabV* than DLBS (*P* < 0.05). DLZX exceeded DLBX in *ruvB*, *mtrD* and *mexF* (*P* < 0.01), and DLZS in *ruvB*, *mtrD* and *sdeB* (*P* < 0.05).

By biocide class, eight categories declined in DLBS, DLBX and DLZS relative to CK (*P* < 0.01): triclosan, dodine, p-xylene, CCCP, TCS, plumbagin, n-hexane and Triton X-100. Three of these, CCCP, TCS and plumbagin, were also lower in DLZX (*P* < 0.001). Triclosan was higher in DLZS than in DLBS (*P* < 0.05). Against DLBX, DLZX showed higher triclosan, dodine, p-xylene, n-hexane and Triton X-100 (*P* < 0.05); against DLZS, higher dodine and Triton X-100 (P < 0.05) (Figures 5A, B).

**FIGURE 5:**
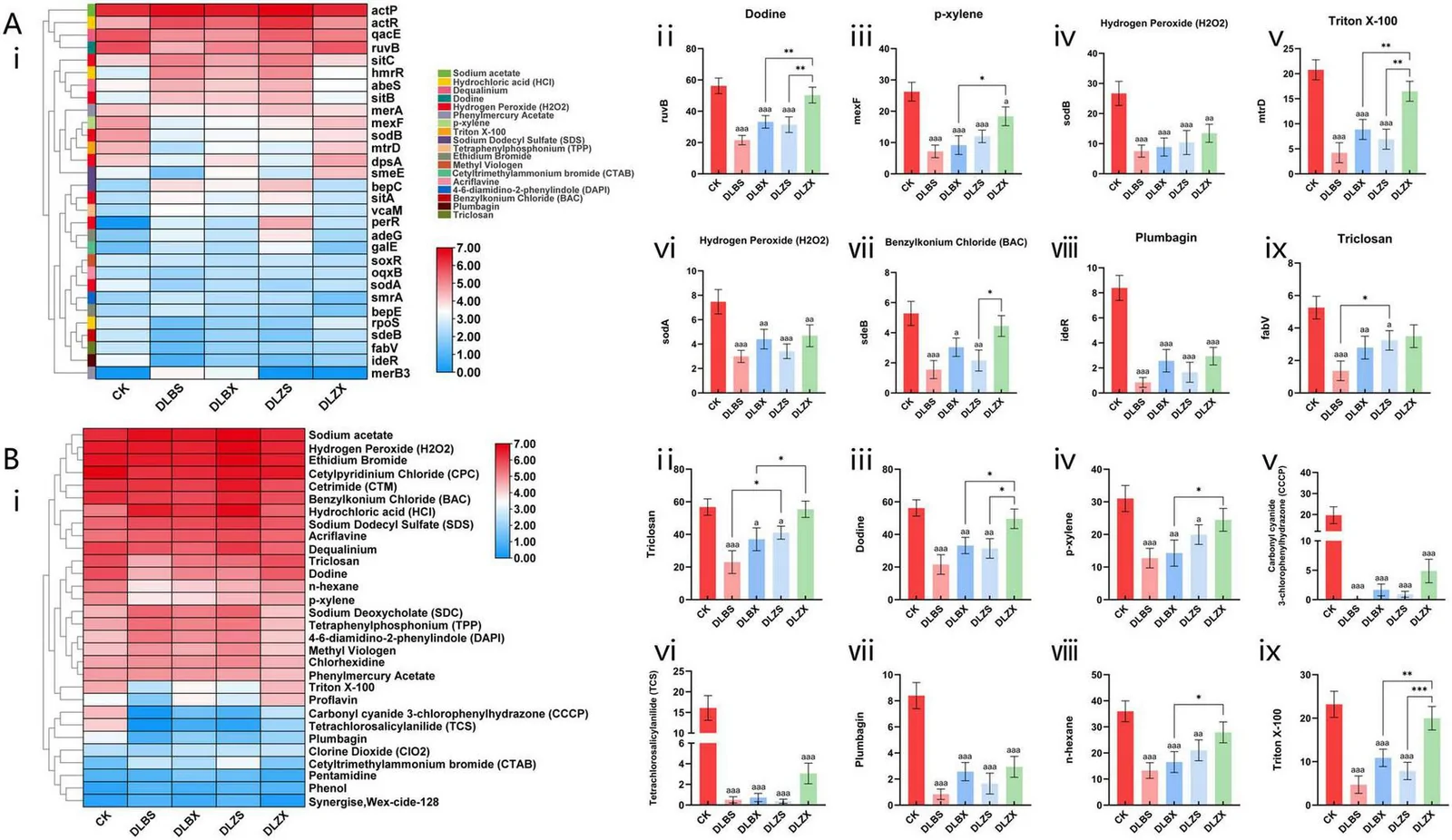
Heatmaps of biocide resistance genes and between-group differences. **(A)** BRG subtypes. **(B)** Biocide classes. *P < 0.05, **P < 0.01, ***P < 0.001.

### Virulence factor genes

Six VFG subtypes, *htpB*, *clpV*, *katA*, *narH*, *sugC* and *glnA1*, were less abundant in DLBS, DLBX and DLZS than in CK (*P* < 0.001), and three of them, *katA*, *narH* and *glnA1*, were also lower in DLZX (*P* < 0.01). DLZX stood out among the fermented treatments. It carried more *htpB*, *clpV*, *narH* and *sugC* than DLBX (*P* < 0.05), and more *htpB*, *clpV* and *sugC* than DLZS (*P* < 0.05) (Figure 6A).

**FIGURE 6:**
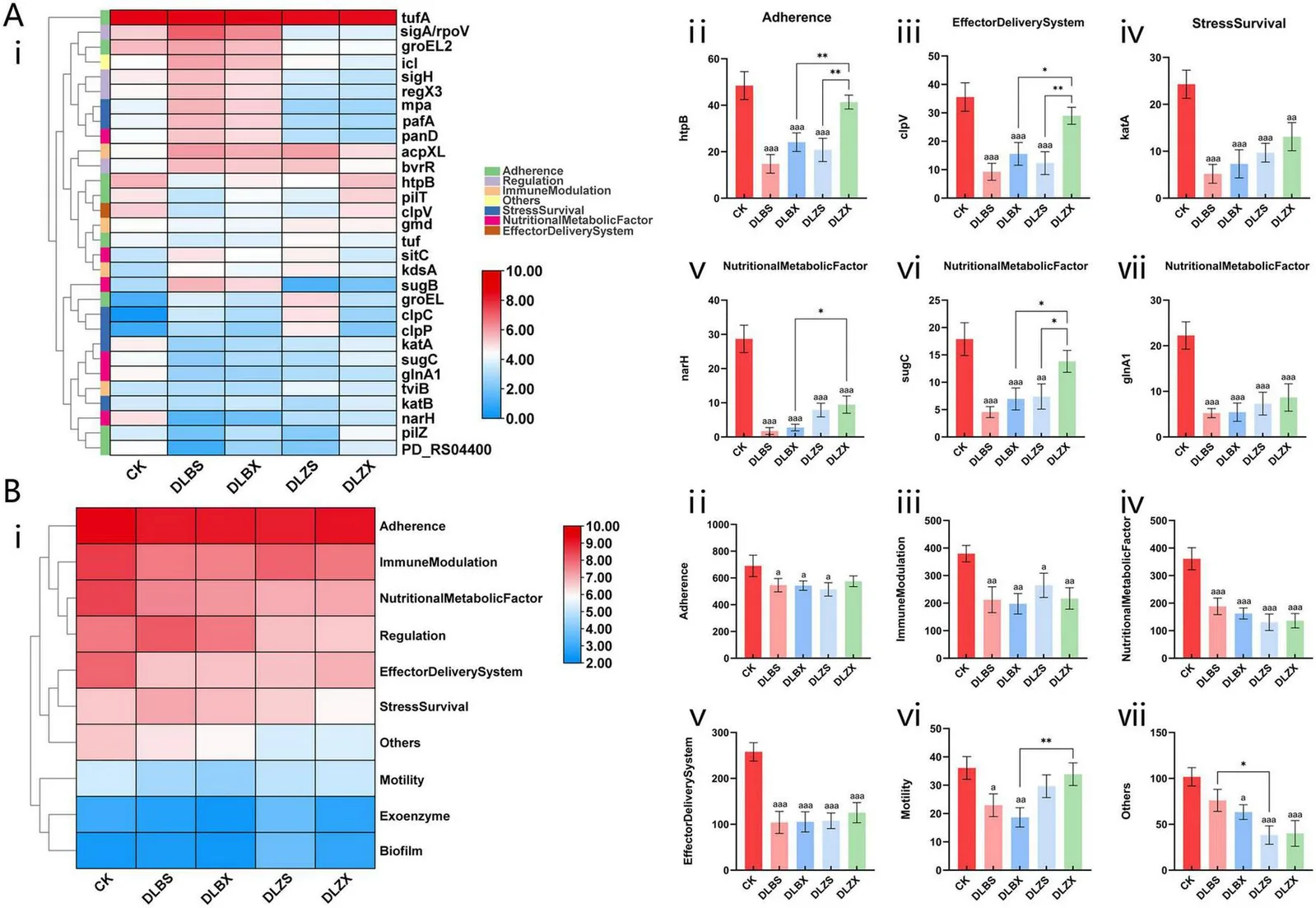
Heatmaps of virulence factor genes and between-group differences. **(A)** VFG subtypes. **(B)** Virulence factor functional categories. *P < 0.05, **P < 0.01, ***P < 0.001.

By functional category, three classes, nutritional and metabolic factor, effector delivery system and immune modulation, were lower in every fermented group than in CK (*P* < 0.01). Adherence fell in DLBS, DLBX and DLZS (*P* < 0.05). The residual “others” category fell in DLBX, DLZS and DLZX (*P* < 0.05), and motility fell in DLBS and DLBX (*P* < 0.05). Between individual pairs, DLZS was depleted in “others” relative to DLBS (*P* < 0.05), and DLZX depleted in motility relative to DLBX (*P* < 0.01) (Figure 6B).

## Discussion

### Fermentation reshapes the litter microbial community and its diversity

Litter from four positions within each of three independent fermentation beds under compound microbial intelligent membrane fermentation was compared with unfermented litter from the same batches for community structure and for the distribution of resistance and virulence determinants. Both the fermentation itself and the position within the bed shaped the composition and function of the litter ecosystem. Because the control was unfermented material of the same origin, the comparison isolates the effect of fermentation itself; because the three beds were operated in parallel as independent units, the treatment effect is not confounded with bed-to-bed differences in management, and the three beds are the biological replicates for every comparison made below. Where the treatment worked, it worked on the genes that matter most for environmental and health risk.

Alpha and beta diversity showed that fermentation did not act uniformly: the edge-upper position (DLBS) had the highest species richness, while the center-lower position (DLZX) stayed closest to unfermented litter. Oxygen gradients inside the bed explain the pattern (Chen et al., 2023; Duan et al., 2023). Air moves more freely through the edge and upper zones, producing a microenvironment better suited to aerobic and facultatively anaerobic microorganisms; the center-lower zone is likely oxygen-limited, so fermentation stalls there. That even a limited oxygen supply can redirect microbial processes in a complex fermentation system is clear from poultry litter treatment, where micro-aeration measurably alters the resistome and virulence gene profile (Agga and Loughrin, 2026). Functional membrane covering conserves heat and improves the pile microenvironment, raising the relative abundance of groups such as the actinomycetes (Ma et al., 2021), and it shifts the bacterial community in winter composting as well (Liu et al., 2026). Because the beds were not turned, the heterogeneity can be attributed to oxygen supply: making fermentation more homogeneous means getting more oxygen into the deeper litter by increasing the air flow through the bottom pipes.

*Bacillota* and *Pseudomonadota* emerged as the dominant phyla, as they do in long-running fermentation beds (Zhang et al., 2015). The genus-level enrichment of thermophiles with well-defined functions, *Planifilum* and *Bacillus* among them, is more informative. A thermophilic microbial consortium added to co-composted sheep and chicken manure litter lengthened the thermophilic phase, raised the abundance and diversity of thermophiles, and left alkali-hydrolysable nitrogen positively correlated with *Bacillus* abundance (Chen et al., 2023). Composting under semi-permeable membrane cover is likewise accelerated by the bacterial succession and organic matter transformation that the membrane imposes (Sun F.-S. et al., 2026), and exogenous additives raised the share of thermophilic aerobes such as thermophilic *Bacillus* during chicken manure–peanut shell composting (Shi et al., 2025). Between them, these observations describe a process that concentrates the functional groups driving organic matter degradation and heat generation.

Potential pathogens declined too, *Escherichia* most visibly, echoing the loss of pathogens as fermentation beds age (Zhang et al., 2015). Competition is the probable mechanism. Thermophilic consortium addition raises the abundance of degraders such as *Streptomyces*, which produce antimicrobial compounds and antagonize pathogens including *E. coli* (Chen et al., 2023); biochar-driven composting develops more complex co-occurrence networks and stronger cooperation among taxa during the thermophilic phase (Duan et al., 2023). Fermentation thus favors beneficial organisms and narrows the niche open to harmful ones.

### Fermentation lowers the abundance of resistance genes and virulence factors

Compound microbial fermentation reduced the abundance of resistance and virulence determinants overall, matching the decline in resistance genes reported for hyperthermophilic composting (Wang et al., 2024; Duan et al., 2026), although the response was not uniform across the four classes or across the four positions.

Antibiotic resistance followed the same direction. Genes conferring resistance to clinically important classes, multidrug and beta-lactams among them, fell overall, as they do under ultra-high temperature and membrane-covered hyperthermophilic composting (Sun et al., 2024; Duan et al., 2026; Li et al., 2026). The ecological basis of such shifts is now coming into focus. Bacteria and viruses jointly drive resistome succession during hyperthermophilic co-composting of cow manure and mushroom residue (Zhou X. et al., 2026), and combined metagenomic and 16S rRNA analysis of dairy manure composting reveals clear temporal coupling between resistance genes and community composition (Zhou X. et al., 2026).

A few genes resisted the trend. *tet(L)* and *fexA* rose in the most active treatments, DLZS and DLBS. Their behavior points to structural remodeling of the ARG pool rather than a uniform proportional decline (Zhou X. et al., 2026), and most plausibly reflects a change in the host reservoir. In raw manure these genes sit largely on mobilisable plasmids in enterococci and *E. coli*, which makes them a high transfer risk. In the fermentation-enriched community, dominated for instance by *Bacillus*, they are more likely chromosomal, and their enrichment can be explained by non-antibiotic selection such as high temperature and acidic metabolites (Werner et al., 2022; Bao et al., 2024). *tet(L)* encodes an efflux pump, so its rise reads most simply as a general stress response of the host cell.

Weighing the two, fermentation reduced the abundance of many of the ARGs that dominate raw manure and that are frequently reported on mobile genetic elements, and this public health gain is likely to outweigh local increases in individual chromosomal genes (Fauszt et al., 2026). Intracellular and extracellular ARGs respond differently to oxytetracycline during swine manure composting (Bao et al., 2024), and resistome composition is tightly linked to community structure in sludge composting (Su et al., 2015). Host tracking based on metagenome-assembled genomes would identify which taxa carry the ARGs that persist, giving a fuller picture of the risks and the benefits (Liu et al., 2024).

Metal resistance responded selectively, in keeping with the principle that community remodeling governs resistance gene dynamics during organic waste treatment (Liu et al., 2021). Genes tied to chromium and mercury, *ruvB* and *merA* among them, were generally depleted. The loss of *merA* should not, however, be read as a reduction in the risk of methylmercury formation. *merA* encodes mercuric reductase, which reduces divalent Hg(II) to volatile elemental Hg(0) and thereby detoxifies mercury; it is the resistance determinant of the mer operon and is not part of a methylation pathway (Barkay et al., 2003). Microbial methylmercury production is governed principally by the *hgcAB* gene pair in anaerobic bacteria such as sulfate-reducing bacteria and methanogens (Parks et al., 2013), and none of the gene classes quantified here reports that capacity. Because Hg(II) is the substrate of both reactions, a weaker reductive sink could in principle leave more rather than less Hg(II) available for methylation, so the direction of the environmental effect cannot be inferred from *merA* abundance alone. What the decline in *merA* does establish is a narrowing of the community’s mercury-detoxification potential. Placing that observation in a risk framework would require profiling *hgcAB* alongside measurements of mercury speciation and methylmercury concentration, which we did not perform here. Bacterial succession is the main driver of metal resistance gene dynamics during manure composting (Xie et al., 2019), and shifts in Cu and Zn bioavailability during composting track the fate of heavy-metal-resistant bacteria and their genes (Li et al., 2023).

Copper and zinc genes behaved oppositely. *copA*, *copP* and *actR* rose sharply in the most active zones. The likeliest reason is that the functional groups fermentation enriches, *Pseudomonas* and *Bacillus* among them, carry metal homeostasis systems as part of their basic physiology. Metagenomic analysis identifies *Pseudomonadota* as the main heavy-metal-resistant group in compost, with *Escherichia*, *Pseudomonas* and *Salmonella* carrying a wide range of heavy metal resistance genes (Li et al., 2023). Upregulation of cop genes may thus be how these organisms cope with the copper entering the litter from feed additives, improving their fitness in the fermentation environment. The pattern is polarized: fermentation suppresses particular high-risk metal resistance genes while reinforcing the community’s capacity to keep essential trace elements in balance.

The balance sheet is mixed. Loss of Hg- and Cr-associated genes is a positive signal. Enrichment of Cu- and As-associated genes in dominant taxa is not, since it raises the possibility that these organisms serve as an MRG reservoir, which matters where background metal concentrations are high. Heavy metal resistance genes often co-occur with ARGs on mobile genetic elements and carry an elevated risk of co-dissemination in animal-derived environments (Su et al., 2015; Li et al., 2023). Pairing these data with measured metal concentrations, and identifying the hosts of the upregulated MRGs and whether they sit on mobile elements, would let the net environmental benefit be judged more precisely.

Biocide resistance fell broadly. Most BRGs declined, including those conferring resistance to quaternary ammonium compounds and triclosan. The likely reason is that reshaping the community removes the disinfectant-tolerant populations widespread in raw litter, some Gram-negative rods among them, and so lowers the background of biological resistance. For farm biosecurity this is a good outcome. Comparable shifts have been reported for biological treatment of poultry litter (Agga and Loughrin, 2026). Membrane covering regulates the process quite comprehensively at industrial scale too, cutting NH3 and greenhouse gas release in dairy manure composting both by inhibiting formation and by blocking emission (Xu et al., 2024).

As with ARGs, however, some BRGs were more abundant in the most active treatment than in the other fermented positions, among them *fabV*, which was higher in DLZS than in DLBS. The genetic background of the dominant fermentation organisms explains this best. *qacE* and *qacEΔ1* commonly co-occur with ARGs on plasmids, integrons and other mobile genetic elements (Su et al., 2015; Wang et al., 2024), and environmental bacteria such as *Pseudomonas* can intrinsically carry multidrug efflux genes such as *mexF* (Li et al., 2023; Liu et al., 2024). The increase in BRGs in DLZS may therefore track the rise of core fermentation organisms such as *Pseudomonas* to dominance, bringing intrinsically encoded resistance genes with it, some of them mobile. That would also explain the higher abundance of triclosan resistance genes in that treatment.

The effect of intelligent membrane fermentation on litter BRGs is thus double-edged. It lowers the broad-spectrum resistance load while selectively reinforcing particular resistance mechanisms as functional groups become dominant. Disinfectant exposure can promote the spread of mobile genetic elements and the co-selection of resistance genes, and this pattern echoes that warning (Wang et al., 2024). Beyond optimizing the process, a more active strategy is to screen candidate strains when preparing the inoculum and to favor those carrying few or none of the high-risk mobile resistance genes such as *qacEdelta1*. The environmental safety benefit is then secured at source.

Virulence genes followed the general decline. Genes linked to stress responses, secretion systems and metabolism, *htpB*, *clpV* and *katA* among them, were consistently depleted, indicating a lower abundance of genes associated with pathogenicity. Metagenomic analysis of thermophilic composting of human excreta likewise found marked shrinkage of both the resistance gene and pathogenicity factor pools (Werner et al., 2022). Interference with pathogen quorum sensing by the dominant fermentation organisms may be involved. The strong enrichment of *Bacillus* (Chen et al., 2023) suggests the functional community can suppress virulence machinery at the molecular level.

Attenuation rather than outright killing aligns with the anti-virulence concept now applied to resistant bacteria (Werner et al., 2022; Agga and Loughrin, 2026). Inhibiting virulence factor expression lowers pathogenicity without imposing strong selection for survival, which should in principle slow the emergence of resistance. Intelligent membrane fermentation may therefore be more than a manure treatment. It is a form of bioengineering that manages biological risk through microbial ecology.

There is direct biosecurity value in monitoring the virulence factor reservoir in litter. The farm environment itself can act as a reservoir of pathogen virulence genes (Agga and Loughrin, 2026; Liu et al., 2024). Effective fermentation substantially compresses that reservoir, which matters both for controlling zoonotic risk at source and for raising hygiene standards in production more generally.

## Limitations and outlook

Three constraints bear on how far these results generalize. Samples came from a single duck farm, so the conclusions need testing across farms of different scale and climate. Metagenomics reports the presence and relative abundance of genes; it cannot separate DNA from living and dead cells, nor demonstrate that transfer occurs. Pairing these data with metabolomics would establish how the selective pressures exerted by antibiotics and heavy metals in the litter actually change, and metatranscriptomics or culturomics would resolve the expression and host range of the key resistance genes.

## Conclusion

Contaminated litter constrains dry-reared duck production, yet fermentation is rarely judged by its effect on the resistome. We compared four bed positions with unfermented litter by shotgun metagenomics. Fermentation enriched beneficial taxa, suppressed potential pathogens, and reduced resistance and virulence determinants, though not uniformly across all classes. A few genes rose in the most active zones, so the reservoir was remodeled rather than purged. This gives intelligent membrane fermentation a quantitative footing as a route to litter with a lower burden of resistance and virulence determinants. The shortfall lies in aeration rather than the inoculant, which gives producers a lever. One farm and DNA-based inference leave open whether the detected genes were carried by living cells.

## Data Availability

The original contributions presented in the study are included in the article/supplementary material, further inquiries can be directed to the corresponding author.
